# A meta-analysis on correlation between interleukin-6 -174G/C polymorphism and end-stage renal disease

**DOI:** 10.1080/0886022X.2017.1281146

**Published:** 2017-02-05

**Authors:** Ye Feng, Yan Tang, Hongwei Zhou, Kaiqing Xie

**Affiliations:** Department of Blood Purification, The First Affiliated Hospital of Guangxi Medical University, Nanning, Guangxi, China

**Keywords:** Interleukin-6, polymorphism, end-stage renal disease, meta-analysis, correlation

## Abstract

**Background:** The level of interleukin-6 (IL-6) and its gene polymorphism are associated with the end-stage renal disease (ESRD) and the related complications. This study aimed to investigate the correction between IL-6 -174G/C polymorphism and ESRD by meta-analysis.

**Methods:** Using the databases including PubMed, Embase, Cochrane library, CNKI, and CBM, the data of case-control studies on correlation between IL-6 -174G/C polymorphism and ESRD from database establishment to January 2016 were collected. According to inclusion and exclusion criteria, the quality of literatures was evaluated. The relevant research data were extracted, followed by meta-analysis using Revman 5.3 software (London, UK). The combined odds ratio (OR) and 95% confidence interval (95%CI) of each genetic model were calculated, and the publication bias data was assessed using the Stata 12.0 software (College Station, TX).

**Results:** A total of five literatures were included, with 1199 cases in case group and 1089 cases in control group. Meta-analysis showed that, there was no significant correlation between each genetic model of IL-6 -174G/C polymorphism and ESRD [(C versus G): OR = 1.36, 95%CI (0.69, 2.66), *p* = .38; (CC + GC versus GG): OR = 1.28, 95%CI (0.58, 2.82), *p* = .54; (CC versus GG + GC): OR = 1.71, 95%CI (0.82, 3.54), *p* = .15; (CC versus GG): OR = 1.74, 95%CI (0.76, 3.99), *p* = .19; (GC versus GG): OR = 1.18, 95%CI (0.55, 2.54), *p* = .67]. The race subgroup analysis showed that, there was no significant correlation between each genetic model of IL-6 -174G/C polymorphism and ESRD in the Caucasians (*p* > .05).

**Conclusion:** IL-6 -174G/C polymorphism has no significant correlation with the susceptibility risk of ESRD, and may not be a risk factor for ESRD.

## Introduction

The onset of chronic kidney disease is insidious, and many patients do not pay enough attention to it. With the development of disease, the chronic kidney disease will eventually progress to the end-stage renal disease (ESRD). Finally, the patients need to rely on renal replacement therapy or renal transplantation to sustain their lives.^1^ However, the progression speed of ESRD varies in different individuals, which may be due to the different genetic susceptibility to disease.^2^ ESRD presents a chronic systemic inflammatory state, and the inflammation may be an important pathophysiological factor in promoting the progression from primary renal disease to ESRD.^3^ ^,^ ^4^ The genetic variation of cytokines can lead to the diversity of immune and inflammatory response. Therefore, studying on the polymorphism of cytokine genes plays an important role in finding the cause of renal failure and the mechanism of disease progression.^5^ It is found that, the level of interleukin-6 (IL-6) and its gene polymorphisms are associated with the ESRD and the related complications.^6–8^ At present, there are some researches on the correlation between the polymorphism of IL-6 gene -174G/C and ESRD. However, due to the small sample size and regional and ethnic differences, the conclusions of the presented researches are controversial. The study carried out a meta-analysis of published case-control studies on polymorphisms of IL-6 gene -174G/C and ESRD. The objective was to explore the correlation between the IL-6 -174G/C polymorphism and the susceptibility to ESRD.

## Materials and methods

### Inclusion and exclusion criteria

The inclusion criteria were as follows: (i) literatures about correlation between the IL-6 -174G/C polymorphism and the risk of ESRD; (ii) containing two groups of subjects, in which the subjects in case group was clinically diagnosed as ESRD, and the subjects in control group were the healthy population unrelated with ESRD; (iii) the data of IL6 -174G/C allele or genotype distribution could be obtained, and there were enough data to calculate the odds ratio (OR) and 95% confidence interval (95%CI); and (iv) the distribution of gene frequency in control group was in line with Hardy–Weinberg equilibrium (HWE).

The exclusion criteria were as follows: (i) reviews, case reports, non-case-control studies; (ii) literatures in which the results did not contain the IL-6 -174G/C polymorphism; (iii) reports in which the genotypes were not complete, the full text or detailed data were not available, or there was no enough data to calculate OR or 95%CI; and (iv) if the data of was repeatedly published in a number of publications, the study of the highest quality was selected.

### Literature retrieval strategy

Database of Pubmed, Embase, Cochrane library, CNKI, and CBM were retrieved, and the retrieval time ranged from database establishment to January 2016. The subject terms included “interleukin-6”, “IL-6”, “end-stage renal disease”, “ESRD” and “polymorphism”. The references attached to the retrieved literature were inquired by hand, aiming to find the literatures which were not included according to above inclusion criteria.

### Literature screening, quality assessment, and data extraction

According to the inclusion and exclusion criteria, two researchers independently conducted the literature screening, quality assessment and data extraction, with the cross checking. The disagreement between two researchers was solved by discussion or by the assistance of the third researcher. According to the evaluation criteria of case-control study in Newcastle Ottawa scale (NOS),^9^ the quality of the included literatures was evaluated. The evaluation criteria included three aspects including selection of research subjects, comparability of research subjects and risk factor exposure. The total score was nine points, and the one with ≥5 points presented the high quality study. The data were extracted according to the data extraction form, which were as follows: first author of literature, publication year, nation and race of research subjects, distribution and number of alleles and genotypes in case group, and whether the gene frequency distribution in control group was consistent with HWE.

### Statistical analysis

*χ* ^2^ test was used to determine whether the gene frequency distribution of the control group was consistent with HWE. *p* < .05 was considered not meeting HWE. Meta-analysis was carried out using Revman 5.3 software (London, UK). The combined OR and 95%CI of five genetic models (allele, dominant model, recessive model, homozygous model, and heterozygous model) were calculated. *χ* ^2^ test was used to evaluate the heterogeneity in included literatures. *p* < .1 or *I* ^2 ^>^ ^50% indicated the presence of heterogeneity among these literatures. The randomized effects model was used for analysis. Otherwise, the fixed effects model was applied. The subgroup analysis was performed based on the population race. The analysis of sensitivity was carried out by evaluating the stability after one-by-one eliminating study. Begg’s and Egger’s tests in Stata 12.0 software (College Station, TX) were used to quantitatively evaluate the publication bias of the results of included literatures, and *p* < .05 indicated the publication bias.

## Results

### Basic characteristics and quality of included literatures

**Figure 1.**
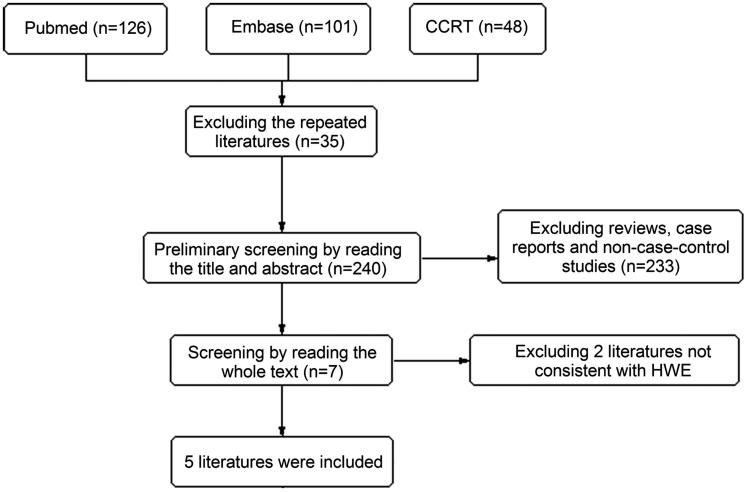
Flowchart on the selection of the literatures.

According to the literature retrieval strategy, 275 literatures were initially obtained. According to the inclusion and exclusion criteria, the repeated literatures, reviews, case reports, and non-case-control literatures were excluded. Seven case-control literatures^10–16^ were included, among which two literatures were excluded after calculating the HWE of gene frequency of control group. Finally, five literatures^12–16^ were remained, including 1199 cases in case group and 1089 cases in control group (Figure 1). The subjects in three literatures were Caucasians, with Africans in one literature and Asians in one literature. The basic characteristics and quality of included literatures were shown in Table 1.

**Table 1.** Basic characteristics and quality of included literatures on correlation between IL-6 -174G/C polymorphism and ESRD.

First author	Year	Race	Country	Case group				Control group				HWE (P)	NOS scores
				GG	GC	CC	N	GG	GC	CC	N		
Buckham^12^	2010	Caucasian	Blended countries	217	299	107	623	194	258	90	542	0.79	6
Kandil^13^	2013	African	Egypt	54	14	2	70	21	8	1	30	0.83	7
Losito^14^	2003	Caucasian	UK	94	57	10	161	105	58	6	169	0.56	7
Ng^15^	2008	Caucasian	USA	83	45	24	152	72	74	22	168	0.66	7
Ranganath^16^	2009	Asian	India	162	80	16	258	515	53	1	569	0.76	6

IL-6: interleukin-6; ESRD: end-stage renal disease; HWE: Hardy–Weinberg equilibrium; NOS: Newcastle Ottawa scale.

### Results of meta-analysis

**Figure 2.**
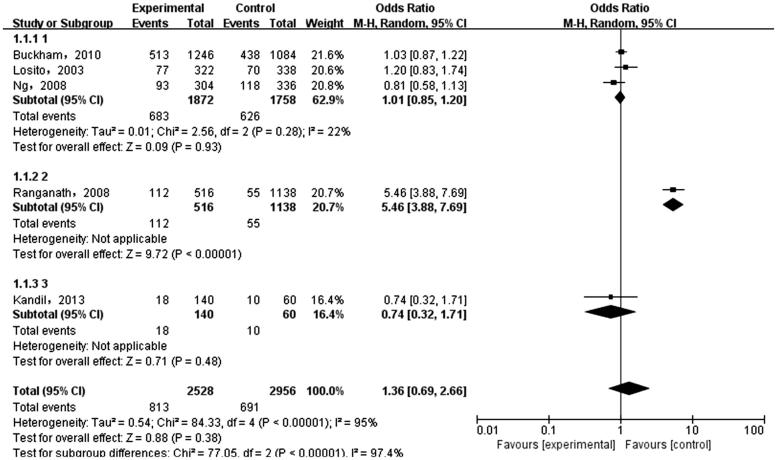
Meta-analysis on correlation between allele (C versus G) and ESRD.

**Figure 3.**
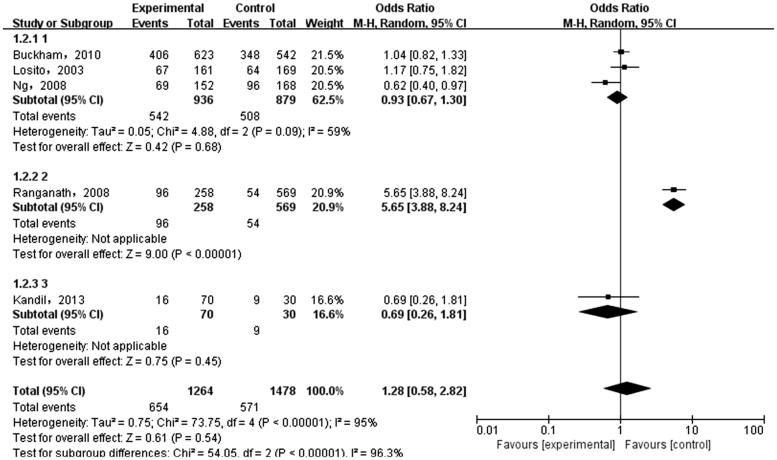
Meta-analysis on correlation between dominant model (CC + CG versus GG) and ESRD.

**Figure 4.**
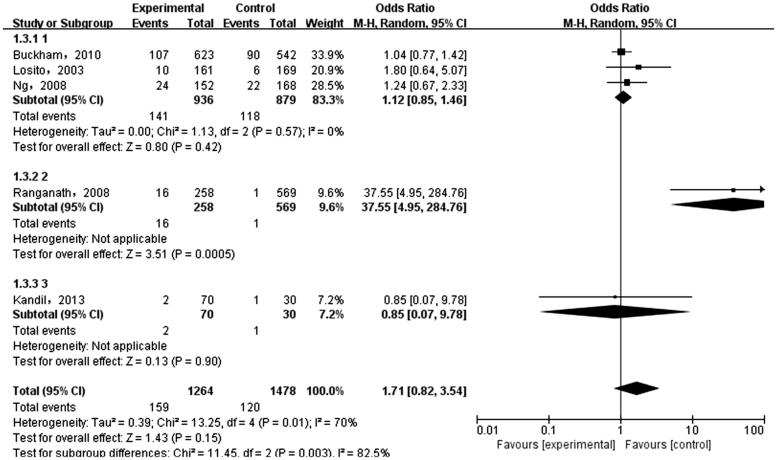
Meta-analysis on correlation between recessive model (CC versus GG + CG) and ESRD.

**Figure 5.**
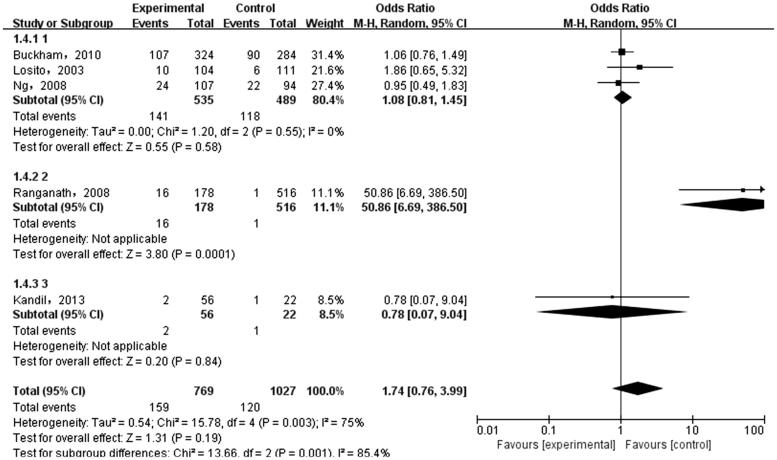
Meta-analysis on correlation between homozygous model (CC versus GG) and ESRD.

**Figure 6.**
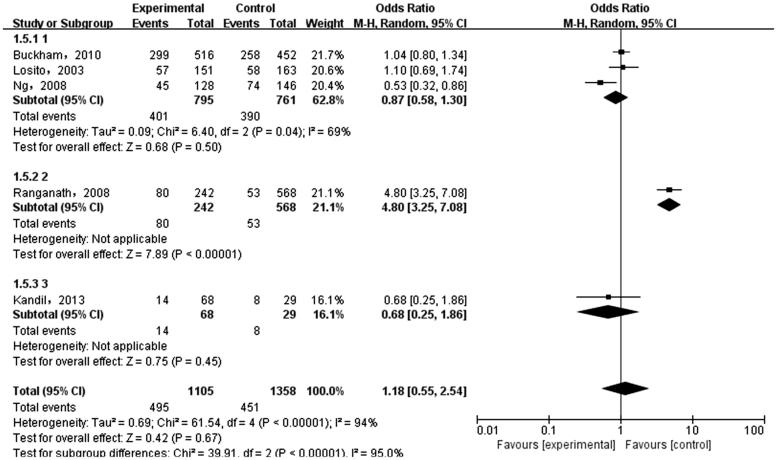
Meta-analysis on correlation between heterozygous model (CG versus GG) and ESRD.

The meta-analysis was performed on the five case-control literatures about the correlation of the IL-6 -174G/C polymorphism and ESRD. Results were shown in Table 2. There was no statistically significant correlation between each genetic model of IL-6 -174G/C polymorphism and ESRD [(C versus G): OR = 1.36, 95%CI (0.69, 2.66), *p* = .38 (Figure 2); (CC + GC versus GG): OR = 1.28, 95%CI (0.58, 2.82), *p* = .54 (Figure 3); (CC versus GG + GC): OR = 1.71, 95%CI (0.82, 3.54), *p* = .15 (Figure 4); (CC versus GG): OR = 1.74, 95%CI (0.76, 3.99), *p* = .19 (Figure 5); (GC versus GG): OR = 1.18, 95%CI (0.55, 2.54), *p* = .67 (Figure 6)].

**Table 2.** Meta-analysis of literatures on correlation between IL-6 -174G/C polymorphism and ESRD in Caucasians.

Subgroup	Genotype comparison	Heterogeneity test		Statistical model	Combined effect		
		I^2^(%)	*p*		OR	95%CI	*p*
Total	C versus G	95	<.01	REM	1.36	0.69–2.66	.38
	CC + GC versus GG	95	<.01	REM	1.28	0.58–2.82	.54
	CC versus GG + GC	70	.01	REM	1.71	0.82–3.54	.15
	CC versus GG	75	<.01	REM	1.74	0.76–3.99	.19
	GC versus GG	94	<.01	REM	1.18	0.55–2.54	.67
Caucasians	C versus G	22	.28	FEM	1.01	0.88–1.16	.86
	CC + GC versus GG	59	.09	REM	0.93	0.67–1.30	.68
	CC versus GG + GC	0	.57	FEM	1.12	0.86–1.46	.42
	CC versus GG	0	.55	FEM	1.09	0.81–1.45	.58
	GC versus GG	69	.04	REM	0.87	0.58–1.30	.50

ESRD: end-stage renal disease; OR: odds ratio; 95%CI: 95% confidence interval; REM: randomized effects model; FEM: fixed effects model.

### Results of race subgroup analysis

The results of the subgroup analysis based on population race showed that, there was no statistically significant correlation between each genetic model in -174G/C polymorphism and ESRD in Caucasians [C versu G): OR = 1.01, 95%CI (0.88, 1.16), *p* = .86 (CC + CG versus; GG: OR = 0.93), 95%CI (0.67, 1.30), *p* = .68 (CC versus GG + CG (0.86): OR = 1.12, 95%CI, 1.46, *p* = .42 (CC); versus GG (OR = 1.09, 95%CI): (0.81, 1.45), *p* = .58 (CG; versus GG): OR = 0.87, 95%CI (0.58, 1.30), *p* = .50] (Table 2).

### Results of sensitivity analysis

The sensitivity analysis was carried out by the method of evaluating the stability after one-by-one eliminating study eliminating single study. There was no significant change in the amount of the combined effects after in turn eliminating 1 literature. This indicated that the results of each literature were stable and reliable.

### Publication bias

**Figure 7.**
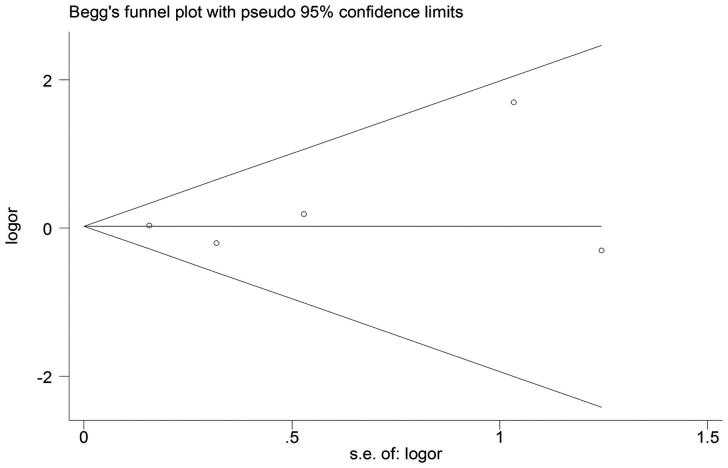
Funnel plot of IL-6 -174G/C polymorphism.

As shown in Figure 7, the scatter distributions in funnel plot of publication bias were symmetrical, with *p* > .05 in both Begg’s and Egger’s test. This indicated that the included literature had no significant publication bias.

## Discussion

ESRD patients are in the state of micro inflammation, which is closely related to vascular sclerosis, anemia, erythropoietin resistance, malnutrition, and infection. These are the important reasons for increased mortality rate of ESRD.^17^ IL-6 is one of the most studied cytokines in chronic kidney disease. The rise of IL-6 levels in ESRD patients is related to the genetic factors (e.g. polymorphisms of single nucleotide), renal function decline, solute retention in uremia, capacity load/chronic heart failure, persistent infection and factors related to dialysis (biocompatibility, dialysis fluid, etc.).^18^

Most of the studies show that, the population carrying G allele in IL-6 -174G/C polymorphism have higher IL-6 level than carrying CC genotype. The G allele can promote the inflammatory response.^13^ ^,^ ^19–21^ Another study shows that, the CC genotype is associated with cardiovascular events in the hemodialysis patients.^6^ Losito et al.^14^ find that, the patients with GC + CC genotype have higher diastolic blood pressure and left ventricular thickness than patients with GG genotype. Ng et al.^15^ find that, the polymorphism of IL-6 -174G/C gene is related to the risk of ESRD. Ranganath et al.^16^ believe that, the frequency of C allele in ESRD patients is significantly higher than that in the control group. Buckham et al.^12^ and Kandil et al.^13^ suggest that, there is no correlation between IL-6 gene polymorphism and ESRD. In this study, five case-control studies are included. In these five studies, the NOS scores are more than five points, so the included literatures have high quality. The meta-analysis results show that, there is no correlation between IL-6 -174G/C polymorphism and ESRD. According to race subgroup analysis, it is found that there is still no correlation between IL-6 -174G/C polymorphism and ESRD in Caucasians.

The results of this study have significant heterogeneity, and the heterogeneity source can be found by subgroup analysis or sensitivity analysis. The subgroup analysis finds that, the heterogeneity is decreased in the studies of allele, recessive model and homozygous model in Caucasians, but the moderate heterogeneity still exists among the dominant model and heterozygous model. In this study, the difference in the characteristics of research subjects, disease diagnostic methods, alleles detection methods, region and environment can lead to the existence of heterogeneity. In this study, by using the one-by-one eliminating method, Ranganath et al.’s study^16^ should be removed, in which the heterogeneity of genetic model is reduced. In addition, in Ranganath et al.’s study,^16^ the subjects are Asians. This indicates that the different races may be the source of heterogeneity. There is no significant change in the re-estimation of the combined effect. The literatures included in this study have no publication bias in statistical significance, which shows that the results of this study are stable and reliable.

This study has some limitations. Firstly, the sample size is relatively small. The race subgroup analysis is only conducted in the Caucasians and the subgroup races. The collected data are not comprehensive. Secondly, parts of the included literatures are lack of indexes such as age, sex, laboratory biochemical indicators in cases and control groups, so the stratified subgroup analysis based on these factors cannot be performed, and the combined results are affected by confounding factors. In addition, the results of this study are not consistent with the results of reported studies. The conclusion still needs to be further confirmed by more large-sample, multi-center, and high-quality case-control studies, for providing a theoretical basis for clarifying the function of IL-6 gene polymorphism in the pathogenesis of ESRD.

## References

[1] Chiou CP, Chung YC. Effectiveness of multimedia interactive patient education on knowledge, uncertainty and decision-making in patients with end-stage renal disease. J Clin Nurs. 2012;21:1223–1231. 21883569 10.1111/j.1365-2702.2011.03793.x

[2] Cox ED, Hoffmann SC, DiMercurio BS, et al Cytokine polymorphic analyses indicate ethnic differences in the allelic distribution of interleukin-2 and interleukin-6. Transplantation. 2001;72:720–726. 11544437 10.1097/00007890-200108270-00027

[3] Akira S, Taga T, Kishimoto T. Interleukin-6 in biology and medicine. Adv Immunol. 1993;54:1–78. 8379461 10.1016/s0065-2776(08)60532-5

[4] Dounousi E, Koliousi E, Papagianni A, et al Mononuclear leukocyte apoptosis and inflammatory markers in patients with chronic kidney disease. Am J Nephrol. 2012;36:531–536. 23258075 10.1159/000345352

[5] Amirzargar AA, Naroueynejad M, Khosravi F, et al Cytokine single nucleotide polymorphisms in Iranian patients with pulmonary tuberculosis. Eur Cytokine Netw. 2006;17:84–89. 16840026

[6] Aker S, Bantis C, Reis P, et al Influence of interleukin-6 G-174C gene polymorphism on coronary artery disease, cardiovascular complications and mortality in dialysis patients. Nephrol Dial Transplant. 2009;24:2847–2851. 19349293 10.1093/ndt/gfp141

[7] Chow KM, Wong TY, Li PK. Genetics of common progressive renal disease. Kidney Int Suppl. 2005;67:S41–S45. 10.1111/j.1523-1755.2005.09409.x 15752238

[8] Stenvinkel P, Pecoits-Filho R, Lindholm B. DialGene Consortium Gene polymorphism association studies in dialysis: the nutrition-inflammation axis. Semin Dial. 2005;18:322–330. 16076356 10.1111/j.1525-139X.2005.18317.x

[9] Stang A. Critical evaluation of the Newcastle-Ottawa scale for the assessment of the quality of nonrandomized studies in meta-analyses. Eur J Epidemiol. 2010;25:603–605. 20652370 10.1007/s10654-010-9491-z

[10] Mittal R, Manchanda P Association of interleukin (IL)-4 intron-3 and IL-6-174 G/C gene polymorphism with susceptibility to end-stage renal disease. Immunogenet Immunogenet. 2007;59:159–165. 10.1007/s00251-006-0182-6 17203290

[11] Sharma R, Agrawal S, Saxena A, Sharma RK. Association of IL-6, IL-10, and TNF-alpha gene polymorphism with malnutrition inflammation syndrome and survival among end stage renal disease patients. J Interferon Cytokine Res. 2013;33:384–391. 23777202 10.1089/jir.2012.0109

[12] Buckham TA, McKnight AJ, Benevente D, Courtney AE, Patterson CC, Simmonds M. Evaluation of five interleukin genes for association with end-stage renal disease in white Europeans. Am J Nephrol. 2010;32:103–108. 20551628 10.1159/000314943

[13] Kandil MH, Magour GM, Khalil GI, Maharem DA, Nomair AM. Possible association of interleukin-1beta (-511C/T) and interleukin-6 (-174G/C) gene polymorphisms with atherosclerosis in end stage renal disease Egyptian patients on maintenance haemodialysis. Egypt. J Med Hum Genet. 2013;14:267–275.

[14] Losito A, Kalidas K, Santoni S, Jeffery S. Association of interleukin-6 -174G/C promoter polymorphism with hypertension and left ventricular hypertrophy in dialysis patients. Kidney Int. 2003;64:616–622. 12846758 10.1046/j.1523-1755.2003.00119.x

[15] Ng DPK, Nurbaya S, Ye SHJ, Krolewski AS. An IL-6 haplotype on human chromosome 7p21 confers risk for impaired renal function in type 2 diabetic patients. Kidney Int. 2008;74:521–527. 18496509 10.1038/ki.2008.202 PMC2756185

[16] Ranganath P, Tripathi G, Sharma RK, Sankhwar SN, Agrawal S. Role of non-HLA genetic variants in end-stage renal disease. Tissue Antigens. 2009;74:147–155. 19497039 10.1111/j.1399-0039.2009.01276.x

[17] Stenvinkel P. Inflammatory and atherosclerotic interactions in the depleted uremic patient. Blood Purif. 2001;19:53–61. 11114578 10.1159/000014479

[18] Suliman ME, Stenvinkel P. Contribution of inflammation to vascular disease in chronic kidney disease patients. Saudi J Kidney Dis Transpl. 2008;19:329–345. 18445891

[19] Burzotta F, Iacoviello L, Di Castelnuovo A, et al Relation of the -174 G/C polymorphism of interleukin-6 to interleukin-6 plasma levels and to length of hospitalization after surgical coronary revascularization. Am J Cardiol. 2001;88:1125–1128. 11703956 10.1016/s0002-9149(01)02046-x

[20] Fernandez-Real JM, Broch M, Vendrell J, Richart C, Ricart W. Interleukin-6 gene polymorphism and lipid abnormalities in healthy subjects. J Clin Endocrinol Metab. 2000;85:1334–1339. 10720087 10.1210/jcem.85.3.6555

[21] Gaudino M, Andreotti F, Zamparelli R, et al The -174G/C interleukin-6 polymorphism influences postoperative interleukin-6 levels and postoperative atrial fibrillation. Is atrial fibrillation an inflammatory complication? Circulation. 2003;108:II195–II199. 12970232 10.1161/01.cir.0000087441.48566.0d

